# Paramagnetic rim lesions lead to pronounced diffuse periplaque white matter damage in multiple sclerosis

**DOI:** 10.1177/13524585231197954

**Published:** 2023-09-15

**Authors:** Nik Krajnc, Victor Schmidbauer, Joel Leinkauf, Lukas Haider, Gabriel Bsteh, Gregor Kasprian, Fritz Leutmezer, Barbara Kornek, Paulus Stefan Rommer, Thomas Berger, Hans Lassmann, Assunta Dal-Bianco, Simon Hametner

**Affiliations:** Department of Neurology, Medical University of Vienna, Vienna, Austria/Comprehensive Center for Clinical Neurosciences and Mental Health, Medical University of Vienna, Vienna, Austria/Faculty of Medicine, University of Ljubljana, Ljubljana, Slovenia; Comprehensive Center for Clinical Neurosciences and Mental Health, Medical University of Vienna, Vienna, Austria/Department of Biomedical Imaging and Image-guided Therapy, Medical University of Vienna, Vienna, Austria; Comprehensive Center for Clinical Neurosciences and Mental Health, Medical University of Vienna, Vienna, Austria/Department of Biomedical Imaging and Image-guided Therapy, Medical University of Vienna, Vienna, Austria; Comprehensive Center for Clinical Neurosciences and Mental Health, Medical University of Vienna, Vienna, Austria/Department of Biomedical Imaging and Image-guided Therapy, Medical University of Vienna, Vienna, Austria; Department of Neurology, Medical University of Vienna, Vienna, Austria/Comprehensive Center for Clinical Neurosciences and Mental Health, Medical University of Vienna, Vienna, Austria; Comprehensive Center for Clinical Neurosciences and Mental Health, Medical University of Vienna, Vienna, Austria/Department of Biomedical Imaging and Image-guided Therapy, Medical University of Vienna, Vienna, Austria; Department of Neurology, Medical University of Vienna, Vienna, Austria/Comprehensive Center for Clinical Neurosciences and Mental Health, Medical University of Vienna, Vienna, Austria; Department of Neurology, Medical University of Vienna, Vienna, Austria/Comprehensive Center for Clinical Neurosciences and Mental Health, Medical University of Vienna, Vienna, Austria; Department of Neurology, Medical University of Vienna, Vienna, Austria/Comprehensive Center for Clinical Neurosciences and Mental Health, Medical University of Vienna, Vienna, Austria; Department of Neurology, Medical University of Vienna, Vienna, Austria/Comprehensive Center for Clinical Neurosciences and Mental Health, Medical University of Vienna, Vienna, Austria; Center for Brain Research, Medical University of Vienna, Vienna, Austria; Department of Neurology, Medical University of Vienna, Vienna, Austria/Comprehensive Center for Clinical Neurosciences and Mental Health, Medical University of Vienna, Vienna, Austria; Comprehensive Center for Clinical Neurosciences and Mental Health, Medical University of Vienna, Vienna, Austria/Division of Neuropathology and Neurochemistry, Department of Neurology, Medical University of Vienna, Vienna, Austria

**Keywords:** Multiple sclerosis, paramagnetic rim lesion, iron, periplaque area, progression, T1 relaxation time, T2 relaxation time, proton density

## Abstract

**Background:**

Paramagnetic rim lesions (PRLs) are an imaging biomarker in multiple sclerosis (MS), associated with a more severe disease.

**Objectives:**

To determine quantitative magnetic resonance imaging (MRI) metrics of PRLs, lesions with diffuse susceptibility-weighted imaging (SWI)-hypointense signal (DSHLs) and SWI-isointense lesions (SILs), their surrounding periplaque area (PPA) and the normal-appearing white matter (NAWM).

**Methods:**

In a cross-sectional study, quantitative MRI metrics were measured in people with multiple sclerosis (pwMS) using the multi-dynamic multi-echo (MDME) sequence post-processing software “SyMRI.”

**Results:**

In 30 pwMS, 59 PRLs, 74 DSHLs, and 107 SILs were identified. Beside longer T1 relaxation times of PRLs compared to DSHLs and SILs (2030.5 (1519–2540) vs 1615.8 (1403.3–1953.5) vs 1199.5 (1089.6–1334.6), both *p* < 0.001), longer T1 relaxation times were observed in the PRL PPA compared to the SIL PPA and the NAWM but not the DSHL PPA. Patients with secondary progressive multiple sclerosis (SPMS) had longer T1 relaxation times in PRLs compared to patients with late relapsing multiple sclerosis (lRMS) (2394.5 (2030.5–3040) vs 1869.3 (1491.4–2451.3), *p* = 0.015) and also in the PRL PPA compared to patients with early relapsing multiple sclerosis (eRMS) (982 (927–1093.5) vs 904.3 (793.3–958.5), *p* = 0.013).

**Conclusion:**

PRLs are more destructive than SILs, leading to diffuse periplaque white matter (WM) damage. The quantitative MRI-based evaluation of the PRL PPA could be a marker for silent progression in pwMS.

## Introduction

Multiple sclerosis (MS) is a chronic inflammatory disease of the central nervous system (CNS), causing demyelinating lesions in the white and gray matter. While acute demyelinating activity is reflected by increased blood–brain barrier (BBB) permeability and subsequent gadolinium (Gd) enhancement, a considerable proportion of chronic active lesions can be depicted by iron accumulation in proinflammatory microglia/macrophages at the lesion edges.^1,2^ These paramagnetic rim lesions (PRLs) allow neuropathologically validated, magnetic resonance imaging (MRI)–based investigation of intra- and peri-plaque tissue damage in MS.^3^ PRLs occur in approximately 60% of people with multiple sclerosis (pwMS) irrespective of their course, peaking in the late relapsing multiple sclerosis (lRMS) and early secondary progressive multiple sclerosis (SPMS).^4^ They expand over time compared to non-PRLs,^3^ indicating persistent inflammation, slow demyelination, and profound tissue destruction.^3,5^ Over several years, iron rims gradually disappear, with the PRL size stabilizing, presumably becoming non-PRLs.^3^ PRLs are considered a relevant biomarker for disease severity, progression, and even earlier conversion to SPMS.^6–8^

To understand faster disease progression in patients with PRLs, the microstructural changes in the PRL periplaque area (PPA) need to be investigated. Neuropathological evaluation of PRLs revealed that the iron rim invariably demarcated the border of the demyelinating lesions. However, the fluid-attenuated inversion recovery (FLAIR)-hyperintense PPAs outside iron rims reflected Wallerian degeneration with reduced axonal density, loosening of the glial matrix, microglia activation, and reactive astrogliosis.^3^ PRLs showed decreased myelin water fraction and neurite density compared to the non-PRLs, with axon and myelin pathology decreasing centrifugally from the lesion core in the PPA regardless of the iron rim presence.^9^ Recently, a more pronounced white matter (WM) damage in the PPA of PRLs versus non-PRLs was also suggested in in vivo studies.^10,11^ Widespread microstructural changes have been also found in the normal-appearing white matter (NAWM) by diffusion-tensor imaging and magnetization transfer ratio studies,^12,13^ probably caused by diffuse accumulation of inflammatory cells and axonal injury, and possibly contributing to disease progression.

Recently, WM lesions with a non-specific diffuse susceptibility-weighted imaging (SWI)-hypointense signal (DSHL) have been described.^14^ The susceptibility changes in these lesions might in part reflect diffuse perivascular iron accumulation, which have been found in MS lesions in a post-mortem MRI–histological correlation study.^15^ These SWI signal changes do not seem to indicate a microglial iron loading indicative of MS-related proinflammatory microglial activation;^16^ however, they might reflect subtle and chronically compromised BBB.

Among quantitative MRI metrics, T1 relaxation times depend on the integrity of micro- and macro-structural tissue components and are prolonged by, for example, demyelination, axonal loss, and oedema.^17^ In early gadolinium (Gd)-enhancing lesions, a prolongation of T1 and T2 relaxation times relates to vasogenic edema, termed acute black hole,^18^ which can be soon followed by shortening of T1 and T2 relaxation times, partly due to partial or even complete remyelination.^19^ Conversely, proton density (PD) primarily reflects the water content in tissue, thus being sensitive to edema and structural damage.

Based on a single quantitative multi-dynamic multi-echo (MDME) sequence, the post-processing tool SyMRI (Synthetic MR AB, Linköping, Sweden) enables the generation of various MRI contrasts, such as T1- and T2-weighted, PD, and inversion recovery,^20^ thereby reducing examination time. Contrary to conventional MRI techniques, repetition time (TR), echo time (TE) and inversion time (TI) are not predefined and are adjustable in retrospect, generating the desired contrasts within a few seconds.

In recent years, highly effective anti-inflammatory disease-modifying therapies (DMTs) have been shown to significantly reduce acute inflammation also by driving down chronic activity,^21,22^ but nevertheless disability in pwMS continues to progress slowly, termed silent progression.^22,23^ Therefore, identification of different subtypes of chronic lesions leading to tissue destruction behind a closed BBB and thereby to insidious clinical progression in pwMS is of interest.^8,24^ This could lead to new therapies against chronic inflammation, which is currently not effectively addressed.

Our aim was to study quantitative MRI metrics of PRLs, DSHLs and SWI-isointense lesions (SILs), their surrounding PPAs and the NAWM in different disease stages to elucidate pathophysiological mechanisms in vivo. We reasoned that PRLs are more destructive than other types of lesions, leading to more pronounced WM damage in their PPA, too.

## Methods

### Patients

**Figure 1.**
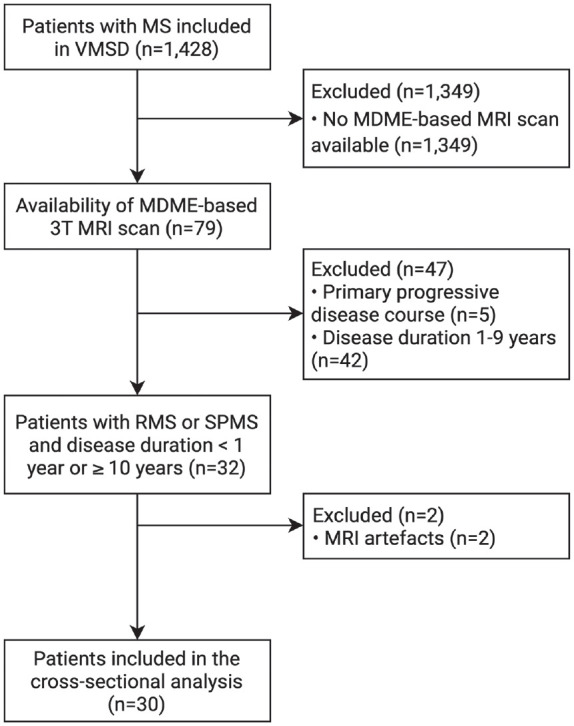
Flow diagram with the inclusion and exclusion criteria.

For this cross-sectional, retrospective study, 30 patients from the Vienna MS database (VMSD)^25^ were included based on the following inclusion criteria: MS diagnosis according to the McDonald criteria valid at the time of diagnosis,^26,27^ age ⩾ 18 years, and availability of T1-, FLAIR-, SWI-, and MDME-based MRI scans at 3T. If more than one MRI scan was available in a patient, the first one in chronological order was selected for the cross-sectional analysis. All MRI scans were performed at least 6 months after the last relapse and/or steroid therapy, and at least 3 months after DMT initiation. The detailed inclusion/exclusion process is depicted in Figure 1. Patients were grouped into early relapsing multiple sclerosis (eRMS) (disease duration ⩽ 1 year), late relapsing multiple sclerosis (lRMS) (disease duration ⩾ 10 years), and clinically definite SPMS with disease duration ⩾10 years.^28–30^ Data on Expanded Disability Status Scale (EDSS) and Multiple Sclerosis Severity Scale (MSSS) according to Roxburgh et al. were obtained at the time of MRI.^31^ DMT status was classified as follows: (1) “no DMT” defined as patients being either treatment-naïve or receiving no DMT at least 6 months prior to the MRI scan; (2) “moderately effective DMT” (M-DMT) defined as patients receiving either interferon-beta, glatiramer acetate, dimethyl fumarate, or teriflunomide; or (3) “highly effective DMT” (H-DMT) defined as patients receiving either natalizumab, fingolimod, siponimod, ponesimod, ozanimod, alemtuzumab, cladribine, ocrelizumab, ofatumumab, or rituximab.

#### Imaging acquisition

All cranial MRI scans were performed on a Philips Ingenia Elition 3.0 T MRI system, using a 32-channel radio frequency (RF) head coil between September 2021 and July 2022. Three-dimensional (3D) FLAIR (1 mm^3^) (TR = 4800 ms, TE = 340 ms, TI = 1650 ms), 3D T2-weighted (1 mm^3^) images (TR = 3000 ms, TE = 280 ms), 3D T1-weighted (0.7 mm^3^) images (TR = 11.43 ms, TE = 5.27 ms) (pre- and post-Gd-based contrast administration), diffusion-weighted imaging (DWI) (TR = 4052.76 ms, TE = 83.22 ms, *b* = 0/1000 s/mm^2^), and SWI sequences (TR = 31 ms, TE = 7.2 ms, matrix = 384 × 315, slices = 143, slice thickness = 2 mm) were acquired consecutively. In addition, an MDME sequence (axial plane) (TR = 4529.15 ms, TE = 100/12.5 ms, matrix = 328 × 219, slices = 30, slice thickness = 4 mm) was acquired, which determines tissue-specific T1-/T2-relaxation properties and PD within a single scan (Supplemental Table 1).^20^ The MDME sequence post-processing software “SyMRI” (Version 11.2.9) was used to generate quantitative maps for region-of-interest (ROI) placement (https://syntheticmr.com/products/symri-neuro/).

### Evaluation of lesions

**Figure 2.**
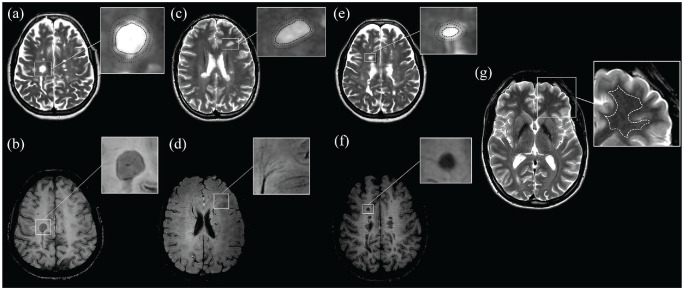
Region-of-interest (ROI) (dashed ROI: relaxometric analysis of the lesion core; dotted ROI: relaxometric analysis of the periplaque area (PPA)) placements are demonstrated on synthetically generated T2-weighted contrasts (repetition time (TR)/echo time (TE): 4500/100 ms) (a, c, and e). The corresponding susceptibility-weighted imaging (SWI) data were used to discriminate paramagnetic rim lesions (PRLs), SWI-isointense lesions (SILs), and lesions with diffuse SWI-hypointense signal (DSHLs) (b, d, and f). A PRL (a and b) with respective ROIs (a) is shown in a 24-year-old male patient with relapsing multiple sclerosis and 0.4 years of disease duration with an EDSS of 0 and an MSSS of 0.67. An SIL (c and d), DSHL (e and f), and the normal-appearing white matter (NAWM) (g) with respective ROIs (c, e, and g) are shown in a 42-year-old female patient with secondary progressive multiple sclerosis and 16.9 years of disease duration with an EDSS of 4.0 and an MSSS of 3.65.

Supratentorial and infratentorial lesions were analyzed in a consensus reading by three raters with experience in neuroradiology (L.H.), clinical neurology (A.D.-B.), and neuropathology (S.H.). PRLs were defined as FLAIR-hyperintense lesions that were partially or completely surrounded by a distinct SWI-hypointense rim spanning a minimum of two consecutive slices in the axial direction (Supplemental Figure S1). Well-demarcated FLAIR-hyperintense lesions without any SWI signal decrease were included as SILs into the analysis (Supplemental Figure S2). FLAIR-hyperintense lesions that showed diffuse SWI signal decrease which neither fulfilled the PRL criteria nor were completely iron negative were termed DSHLs (Supplemental Figure S3).^14^ Gd-enhancing lesions were excluded from the analysis (*n* = 2). MRI metrics (T1 and T2 relaxation times, PD) were measured on quantitative, “SyMRI”-generated maps in all identified PRLs, DSHLs, and SILs, their corresponding PPAs, and NAWM (Figure 2). ROI placement was performed manually on quantitative, MDME-based imaging data. The MDME sequence determines the relaxation time metrics and the PD values via two repeated phases of acquisition: phase I: a 120° saturation pulse is applied to saturate one section, and phase II: excitation pulses (90°) and refocusing pulses (180°) are applied to generate echo trains.^20,32,33^ The post-processing software SyMR^®^ was used to determine the quantitative properties, providing mean values of T1-/T2-relaxation times and PD metrics within the drawn ROI. For NAWM, ROI drawings were performed centrally in the deep WM of the frontal, temporal, or parietal lobe (periventricular ROI placement and ROI drawings near lesion location were avoided). For all types of lesions, interspaces between intra- and peri-lesional ROIs were maintained. The distance between the ROI border for the lesion core and the ROI border for the respective PPA was evaluated qualitatively. The manually drawn ROIs were cautiously placed with a distance between the borders of the lesion and anatomical borders in order to avoid partial volume effects. Only lesions with adequate perilesional space/WM for ROI placement were included. Confluent lesions were integrated for analysis, provided the lesions were well demarcated. To reduce potential measurement biases, two measurements were performed either on the same slice or, if available, on two contiguous slices, and the mean value of both was used for further analyses. The brain parenchymal volumes (BPVs) were automatically calculated based on the relaxometric features within the intracranial voxels that were assigned to cerebral parenchyma.

### Ethics

The study was approved by the ethics committee of the Medical University of Vienna (EC 1378/2020 and 1380/2020). The requirement for written informed consent from study participants was waived by the ethics committee (retrospective study).

### Statistics

Statistical analysis was performed using SPSS 26.0 (SPSS Inc., Chicago, IL, USA). Categorical variables were expressed in frequencies and percentages, continuous variables as mean value and standard deviation (SD) or median and interquartile range (IQR) as appropriate. Continuous variables were tested for normal distribution by the Kolmogorov–Smirnov test with the Lilliefors correction. Univariate comparisons were done by the chi-square test, one-way analysis of variance (ANOVA), and the Kruskal–Wallis test with pairwise comparisons. Univariate correlation analyses were performed on MRI metrics with clinical and paraclinical data (age, disease duration, EDSS, MSSS, BPV) using the Pearson or Spearman test as appropriate. A value of *p* < 0.05 was considered statistically significant. All multiple analyses were corrected using the Bonferroni method.

## Results

A total of 59 PRLs, 74 DSHLs, and 107 SILs were identified in 30 patients (median age 39.5 years (SD = 11.0), 73.3% female, median EDSS 2.3 (range = 0–6.0)). Median number of PRLs per patient was 1 (range = 0–10), with 13 (43.3%) patients having no PRLs. Demographics of the study cohort are presented in Table 1.

**Table 1.** Clinical characteristics of the study cohort.

	All (*n* = 30)	eRMS (*n* = 10)	lRMS (*n* = 11)	SPMS (*n* = 9)	*p*
Demographics and clinical characteristics					
Female^a^	22 (73.3)	5 (50.0)	10 (90.9)	7 (77.8)	0.100^e^
Age (years)^b^	**39.5 (11.0)**	**32.0 (6.2)**	**37.4 (9.9)**	**50.6 (7.6)**	**<0.001** ^f^
Disease duration (years)^c^	**12 (0.6–16.4)**	**0.4 (0.3–0.6)**	**13.9 (11.3–17.5)**	**15.3 (13.0–23.5)**	**<0.001** ^g^
EDSS^d^	**2.3 (0–6.0)**	**1.0 (0–2.0)**	**2.5 (0–4.0)**	**4.0 (3.0–6.0)**	**<0.001** ^g^
MSSS^c^	3.09 (0.10–7.39)	3.27 (0.67–5.87)	1.48 (0.10–4.26)	3.15 (2.71–7.39)	0.300^g^
DMT^a,h^					
No DMT	5 (16.7)	1 (10.0)	2 (18.2)	2 (22.2)	0.575^e^
M-DMT	8 (26.7)	4 (40.0)	1 (9.1)	3 (33.3)	
Interferon	1 (3.3)	0 (0.0)	0 (0.0)	1 (11.1)	
Glatiramer acetate	2 (6.7)	1 (10.0)	1 (9.1)	0 (0.0)	
Dimethyl fumarate	4 (13.3)	3 (30.0)	0 (0.0)	1 (11.1)	
Teriflunomide	1 (3.3)	0 (0.0)	0 (0.0)	1 (11.1)	
H-DMT	16 (53.3)	5 (50.0)	7 (63.9)	4 (44.4)	
Cladribine	3 (10.0)	0 (0.0)	2 (18.2)	1 (11.1)	
Natalizumab	2 (6.7)	1 (10.0)	1 (9.1)	0 (0.0)	
S1PRM	7 (23.3)	3 (30.0)	1 (9.1)	3 (33.3)	
Anti-CD20 mAbs	4 (13.3)	1 (10.0)	3 (27.3)	0 (0.0)	
MRI data					
Presence of PRLs^a^	17 (56.7)	6 (60.0)	7 (63.6)	4 (44.4)	0.667^e^
Number of PRLs^d^	1 (0–10)	1 (0–5)	2 (0–10)	0 (0–4)	0.475^g^
BPV (mL)^b^	**1169.9 (134.4)**	**1266.4 (102.1)**	**1115.0 (143.2)**	**1129.9 (102.9)**	**0.015** ^f^

BPV: brain parenchymal volume; CD: cluster of differentiation; DMT: disease-modifying treatment; EDSS: Expanded Disability Status Scale; H-DMT: highly effective DMT; mAb: monoclonal antibody; M-DMT: moderately effective disease-modifying treatment; MSSS: Multiple Sclerosis Severity Scale; PRL: paramagnetic rim lesion; MRI: magnetic resonance imaging; S1PRM: sphingosine-1-phosphate receptor modulator.

a Number (percentage).

b Mean (standard deviation).

c Median (interquartile range).

d Median (range).

e Chi-square test.

f One-way analysis of variance (ANOVA).

g Kruskal–Wallis test

h One patient’s data is missing.

Bold values denote statistical significance at the *p* < 0.05 level.

## Cross-sectional results

### T1 relaxation time

**Figure 3.**
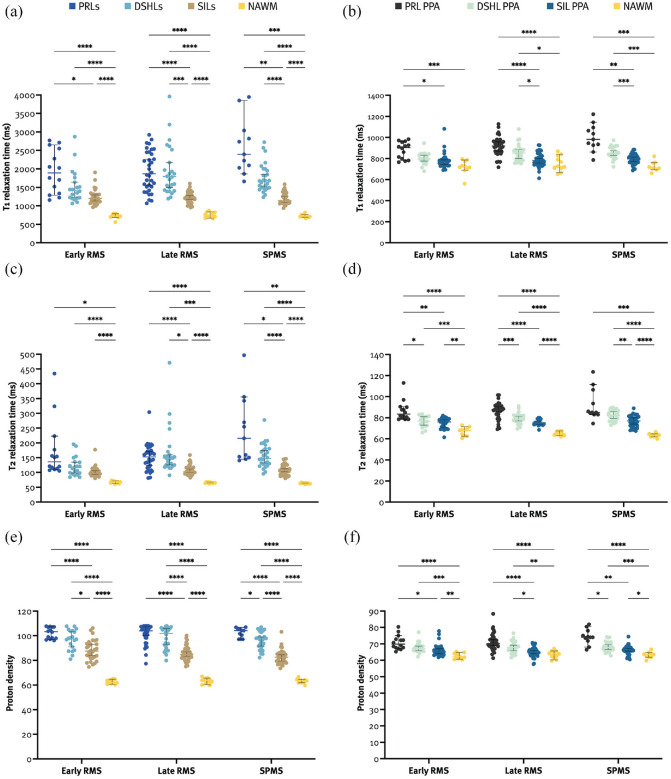
Quantitative MRI metrics of PRLs were longer than those of SILs and the NAWM (a, c, and e). The PRL PPA had longer quantitative MRI metrics than the SIL PPA and the NAWM (b, d, and f). DSHL: lesion with diffuse SWI-hypointense signal; NAWM: normal-appearing white matter; PD: proton density; PPA: periplaque area; PRL: paramagnetic rim lesion; RMS: relapsing multiple sclerosis; SIL: SWI-isointense lesion; SPMS: secondary progressive multiple sclerosis. *p < 0.05; **p < 0.01; ***p < 0.001; ****p < 0.0001.

Overall, PRLs had longer T1 relaxation times than DSHLs and SILs (2030.5 (1519–2540) vs 1615.8 (1403.3–1953.5) vs 1199.5 (1089.6–1334.6), both *p* < 0.001), and DSHLs had longer T1 relaxation times than SILs (*p* < 0.001). The PRL PPA had longer T1 relaxation times than the SIL PPA and the NAWM (926.5 (860.5–980.5) vs 780.3 (748.4–814.9) vs 723.8 (706.9–787.3), both *p* < 0.001) but not the DSHL PPA (835.8 (793–873.9), *p* = 0.621). Patients with SPMS had longer T1 relaxation times in PRLs compared to patients with lRMS (2394.5 (2030.5–3040) vs 1869.3 (1491.4–2451.3), *p* = 0.015), and also in the PRL PPA compared to patients with eRMS (982 (927–1093.5) vs 904.3 (793.3–958.5), *p* = 0.013). Also, patients with eRMS had significantly shorter T1 relaxation times in the DSHL PPA compared to patients with lRMS (796.5 (773.6–841.3) vs 851.8 (793.9–885.9), *p* = 0.033) and SPMS (845.8 (822.6–903.9), *p* = 0.021) (Supplemental Table 2). More importantly, T1 relaxation times in the PRL PPA were significantly longer compared to the SIL PPA and NAWM in all groups, whereas no differences in the PRL PPA and the DSHL PPA were found (Figure 3 and Supplemental Table 2).

### T2 relaxation time

Overall, PRLs had longer T2 relaxation times than SILs (145 (120–192) vs 102 (93.3–113.4), *p* = 0.001) but not DSHLs (136.8 (118.9–169.6), *p* > 0.999). The PRL PPA had longer T2 relaxation times than the SIL PPA and the NAWM (87 (81.5–91.5) vs 76.5 (72.5–80) vs 64.8 (63–67.5), both *p* < 0.001) but not the DSHL PPA (79.8 (76.4–83.1), *p* = 0.080). Patients with SPMS had longer T2 relaxation times in PRLs compared to lRMS (215.5 (150.5–342.5) vs 136 (112–188.5), *p* = 0.019). Also, patients with eRMS had significantly shorter T2 relaxation times in DSHLs compared to patients with lRMS (116.5 (95.8–135.6) vs 148.5 (124.1–174.5), *p* = 0.015) and SPMS (147.8 (128.5–180.4), *p* < 0.001), and in the DSHL PPA compared to patients with SPMS (76.8 (72.6–81) vs 82 (77.6–86.5), *p* < 0.001) (Supplemental Table 2). More importantly, T2 relaxation times in the PRL PPA were significantly longer compared to the NAWM in all groups, and also compared to the DSHL PPA and the SIL PPA in eRMS and lRMS (Figure 3 and Supplemental Table 2).

### PD

Overall, PRLs had higher PD metrics than SILs (103.6 (98.2–107.1) vs 84.3 (80.7–89.3), *p* < 0.001) but not DSHLs (97.8 (89.9–104.6), *p* > 0.999). The PRL PPA had higher PD metrics than the SIL PPA and the NAWM (70.7 (68.0–74.2) vs 65.7 (63.8–67.3) vs 63.1 (61.5–64.4), both *p* < 0.001) but not the DSHL PPA (67.4 (65.7–69.7), *p* = 0.574). Patients with SPMS had significantly lower PD metrics in SILs compared to eRMS (82.6 (79–86) vs 87.5 (81.9–93.9), *p* = 0.021) (Supplemental Table 2). More importantly, PD metrics in the PRL PPA were significantly higher compared to the SIL PPA and the NAWM in all groups and also compared to the DSHL PPA in patients with SPMS (Figure 3 and Supplemental Table 2). Taken together, all MRI metrics point in the same direction, providing complementary results that underpin each other.

### MRI metrics and clinical data

T1-measured neuroaxonal damage around DSHLs (DSHL PPA) was found to correlate with disease duration (*r_s_* = 0.335, *p* = 0.017), EDSS (*r_s_* = 0.312, *p* = 0.038), and BPV (*r_s_* = −0.394, *p* = 0.003), and the T1 relaxation times in the SIL PPA positively correlated with EDSS (*r_s_* = 0.255, *p* = 0.048), whereas no significant correlations between PRLs, the PRL PPA, and clinical data were found. By contrast, T2 relaxation times in DSHLs and their corresponding PPA positively correlated with age (*r_s_* = 0.333, *p* = 0.019; *r_s_* = 0.406, *p* = 0.002), disease duration (*r_s_* = 0.360, *p* = 0.008; *r_s_* = 0.446, *p* < 0.001) and EDSS (*r_s_* = 0.348, *p* = 0.014; *r_s_* = 0.328, *p* = 0.024), respectively, and T2 relaxation times in SILs positively correlated with age (*r_s_* = 0.336, *p* = 0.002) and negatively correlated with BPV (*r_s_* = −0.259, *p* = 0.037). Also, PD in SILs negatively correlated with age (*r_s_* = −0.378, *p* < 0.001), disease duration (*r_s_* = −0.319, *p* = 0.004) and EDSS (*r_s_* = −0.268, *p* = 0.033), and positively with BPV (*r_s_* = 0.351, *p* = 0.001). Additional correlations of MRI metrics with demographical and clinical data are shown in Table 2.

**Table 2.** Univariate analyses of MRI metrics and clinical and paraclinical data.

	Age	Disease duration	EDSS	MSSS	BPV
T1 relaxation time					
PRLs (*n* = 59)	*r_s_* = 0.049 (*p* > 0.999)	*r_s_* = 0.250 (*p* = 0.281)	*r_s_* = 0.176 (*p* = 0.917)	*r_s_* = −0.048 (*p* > 0.999)	*r_s_* = 0.034 (*p* > 0.999)
PRL PPA (*n* = 59)	*r_s_* = 0.111 (*p* > 0.999)	*r_s_* = 0.323 (*p* = 0.062)	*r_s_* = 0.137 (*p* > 0.999)	*r_s_* = −0.142 (*p* > 0.999)	*r_s_* = −0.053 (*p* > 0.999)
DSHLs (*n* = 74)	*r_s_* = 0.235 (*p* = 0.217)	*r_s_* = 0.237 (*p* = 0.212)	*r_s_* = 0.275 (*p* = 0.098)	*r_s_* = −0.020 (*p* > 0.999)	*r_s_* = −0.237 (*p* = 0.212)
DSHL PPA (*n* = 74)	*r_s_* = 0.293 (*p* = 0.057)	***r_s_*** **=** **0.335 (***p* **=** **0.017)**	***r_s_*** **=** **0.312 (***p* **=** **0.038)**	*r_s_* = 0.052 (*p* > 0.999)	***r_s_*** **=** **−0.394 (***p* **=** **0.003)**
SILs (*n* = 107)	*r_s_* = −0.020 (*p* > 0.999)	*r_s_* = −0.079 (*p* > 0.999)	*r_s_* = 0.003 (*p* > 0.999)	*r_s_* = 0.028 (*p* > 0.999)	*r_s_* = 0.075 (*p* > 0.999)
SIL PPA (*n* = 107)	*r_s_* = 0.151 (*p* = 0.595)	*r_s_* = 0.200 (*p* = 0.197)	***r_s_*** **=** **0.255 (***p* **=** **0.048)**	*r_s_* = 0.177 (*p* = 0.372)	*r_s_* = −0.105 (*p* > 0.999)
NAWM (*n* = 30)	*r_s_* = −0.121 (*p* > 0.999)	*r_s_* = 0.074 (*p* > 0.999)	*r_s_* = −0.314 (*p* = 0.521)	*r_s_* = −0.457 (*p* = 0.073)	*r_s_* = −0.071 (*p* > 0.999)
T2 relaxation time					
PRLs (*n* = 59)	*r_s_* = 0.054 (*p* > 0.999)	*r_s_* = 0.324 (*p* = 0.061)	*r_s_* = 0.112 (*p* > 0.999)	*r_s_* = −0.148 (*p* > 0.999)	*r_s_* = 0.121 (*p* = 0.118)
PRL PPA (*n* = 59)	*r_s_* = 0.085 (*p* > 0.999)	*r_s_* = 0.156 (*p* > 0.999)	*r_s_* = −0.146 (*p* > 0.999)	*r_s_* = −0.317 (*p* = 0.073)	*r_s_* = 0.211 (*p* = 0.543)
DSHLs (*n* = 74)	***r_s_*** **=** **0.333 (***p* **=** **0.019)**	***r_s_*** **=** **0.360 (***p* **=** **0.008)**	***r_s_*** **=** **0.348 (***p* **=** **0.014)**	*r_s_* = 0.013 (*p* > 0.999)	*r_s_* = −0.295 (*p* = 0.054)
DSHL PPA (*n* = 74)	***r_s_*** **=** **0.406 (***p* **=** **0.002)**	***r_s_*** **=** **0.446 (***p* **<** **0.001)**	***r_s_*** **=** **0.328 (***p* **=** **0.024)**	*r_s_* = −0.080 (*p* > 0.999)	*r_s_* = −0.254 (*p* = 0.116)
SILs (*n* = 107)	***r_s_* =** **0.336 (***p* **=** **0.002)**	*r_s_* = 0.188 (*p* = 0.269)	*r_s_* = 0.201 (*p* = 0.218)	*r_s_* = 0.089 (*p* > 0.999)	***r_s_*** **=** **−0.259 (***p* **=** **0.037)**
SIL PPA (*n* = 107)	*r_s_* = 0.237 (*p* = 0.073)	*r_s_* = 0.108 (*p* > 0.999)	*r_s_* = 0.138 (*p* = 0.844)	*r_s_* = 0.089 (*p* > 0.999)	*r_s_* = −0.194 (*p* = 0.232)
NAWM (*n* = 30)	*r_s_* = −0.302 (*p* = 0.526)	*r_s_* = −0.262 (*p* = 0.811)	*r_s_* = −0.388 (*p* = 0.206)	*r_s_* = −0.233 (*p* > 0.999)	*r_s_* = 0.201 (*p* > 0.999)
PD metrics					
PRLs (*n* = 59)	*r_s_* = −0.089 (*p* > 0.999)	*r_s_* = −0.042 (*p* > 0.999)	*r_s_* = 0.036 (*p* > 0.999)	*r_s_* = 0.036 (*p* > 0.999)	*r_s_* = −0.064 (*p* = 0.855)
PRL PPA (*n* = 59)	*r_s_* = 0.047 (*p* > 0.999)	*r_s_* = 0.097 (*p* > 0.999)	*r_s_* = 0.058 (*p* > 0.999)	*r_s_* = −0.081 (*p* > 0.999)	*r_s_* = 0.097 (*p* > 0.999)
DSHLs (*n* = 74)	*r_s_* = −0.084 (*p* > 0.999)	*r_s_* = −0.065 (*p* > 0.999)	*r_s_* = 0.072 (*p* > 0.999)	*r_s_* = 0.048 (*p* > 0.999)	*r_s_* = −0.031 (*p* > 0.999)
DSHL PPA (*n* = 74)	*r_s_* = 0.106 (*p* > 0.999)	*r_s_* = 0.097 (*p* > 0.999)	*r_s_* = 0.202 (*p* = 0.445)	*r_s_* = 0.100 (*p* > 0.999)	*r_s_* = −0.251 (*p* = 0.154)
SILs (*n* = 107)	***r_s_*** **=** **−0.378 (***p* **<** **0.001)**	***r_s_*** **=** **−0.319 (***p* **=** **0.004)**	***r_s_*** **=** **−0.268 (***p* **=** **0.033)**	*r_s_* = −0.127 (*p* > 0.999)	***r_s_*** **=** **0.351 (***p* **=** **0.001)**
SIL PPA (*n* = 107)	*r_s_* = 0.043 (*p* > 0.999)	*r_s_* = 0.024 (*p* > 0.999)	*r_s_* = 0.172 (*p* = 0.429)	*r_s_* = 0.181 (*p* = 0.351)	*r_s_* = −0.051 (*p* > 0.999)
NAWM (*n* = 30)	*r_s_* = 0.190 (*p* > 0.999)	*r_s_* = 0.145 (*p* > 0.999)	*r_s_* = 0.020 (*p* > 0.999)	*r_s_* = −0.183 (*p* > 0.999)	*r_s_* = −0.264 (*p* = 0.794)

BPV: brain parenchymal volume; EDSS: Expanded Disability Status Scale; DSHL: diffuse SWI-hypointense signal; MSSS: Multiple Sclerosis Severity Scale; NAWM: normal-appearing white matter; PD: proton density; PPA: periplaque area; PRL: paramagnetic rim lesion; RMS: relapsing multiple sclerosis; SIL: SWI-isointense lesion; SPMS: secondary progressive multiple sclerosis.

Bold values denote statistical significance at the *p* < 0.05 level.

## Discussion

In this retrospective, cross-sectional study, we assessed the MRI metrics in PRLs, DSHLs and SILs, their surrounding PPAs, and the NAWM distant from any lesions. To our knowledge, this is the first study quantifying the MRI characteristics of the above-mentioned lesions in different MS phases. Two novel and key findings arise from this study: (1) PRLs lead to a more pronounced periplaque WM damage compared to SILs but not DSHLs and (2) T1-measured neuroaxonal damage of PRLs and DSHLs, and their corresponding PPA is more pronounced in later disease stages in our data, since T1 relaxation times in these ROIs were significantly longer in patients with SPMS compared to patients with eRMS and/or lRMS.

PRLs have been already shown to display more severe tissue destruction in their center than non-PRLs, reflected by stronger T1 hypointensity,^6,9,14^ and lower myelin water fraction and neurite density index of lesion cores.^34^ Higher brain atrophy rates,^35^ alongside with elevated serum neurofilament levels (sNfLs)^5,35^ and lower retinal layer thickness^36^ in patients with PRLs, indicate that the latter are overall a very useful surrogate marker for increased neurodegeneration in pwMS. Our study provides additional insights into PRL pathophysiology, showing that the neurodegenerative process is not only limited to PRLs themselves but also leads to a more pronounced axonal loss in their PPA.

A plausible mechanism underlying a more pronounced WM damage around PRLs is Wallerian degeneration and secondary demyelination in the surrounding tissue, which is at least partially driven by an ongoing low-grade inflammation at the edges of PRLs.^3^ Iron-laden microglia/macrophages display a proinflammatory phenotype that contributes to edge-related chronic inflammation in those lesions.^1^ These lesion edges also show increased numbers of amyloid precursor protein-positive axonal transections,^5^ which typically lead to subsequent degeneration of the whole axon and neuron, that is, the potential of non-local tissue effects along WM tracts traversing the lesions.Nevertheless, SILs do show a certain increase in T1 and T2 relaxation times in their corresponding PPAs, albeit to a lesser extent. A major proportion of chronic SILs lack activated microglia/macrophages at their edge and thus represent inactive or even partially remyelinated lesions.^1^ Remyelination has been shown to protect against axonal degeneration, and chronic inactive lesions displayed less axonal degeneration than slowly expanding rim lesions.^37^ These data may explain the less destructive potential of SILs, which is reflected by their lower extent of periplaque changes in MRI metrics shown here. Recently, a similar study using T1/T2-weighted ratio to assess tissue integrity in PRLs and their corresponding PPA showed that the PRL PPA indeed exhibits more pronounced tissue damage.^11^

We did not find any correlations between MRI metrics in PRLs and the PRL PPA and clinical data, with some correlations having been found in the DSHL PPA and SILs. However, we show that T1 relaxation times in PRLs and DSHLs and their corresponding PPA are significantly longer in later disease stages, suggesting that changes in those lesions might indeed be dynamic. Also, PRLs had significantly longer T1 relaxation times compared to DSHLs, which stresses out the importance of distinction between those two types of lesions, also because DSHLs show some degree of shrinkage whereas PRLs tend to enlarge over time, and as lesions with a hypointense core detected on SWI-sequence do not necessarily reflect the T1-weighted hypointense lesions (i.e. black holes).^14^

These findings are highly important, since the periplaque changes due to rim inflammation and Wallerian degeneration could explain in part the pathophysiological mechanisms behind the silent disease progression in MS. Periplaque changes of PRLs might in that way turn out to be the most relevant tissue biomarker for disability progression in patients with PRLs for three reasons: (1) the actual expansion of the PRL rim is too slow and partially counteracted by tissue shrinkage due to continuous axonal degeneration within the PRLs resulting in important time loss for the patient; (2) neuropathologically, the amount of axonal degeneration at PRL rims correlates well with the presence of activated microglia/macrophages at the rim, suggesting that MRI-detectable Wallerian degeneration around PRLs likely correlate, too; and (3) these periplaque changes in quantitative MRI metrics can be feasibly assessed, if combined with an iron-sensitive sequence for paramagnetic rim detection.

There are some limitations to this study, including the cross-sectional study design and a small yet clinically well-characterized cohort. Besides, due to the lack of consensus on the radiological definition of PRL, PRLs could have been over- or under-represented in our study, which makes our approach less suitable for estimating their prevalence yet still seems optimal for quantifying tissue destruction in different types of lesions. Also, we did not incorporate clinical activity in the exclusion process, although this drawback was partially overcome by performing MRI scans at least 6 months after the last relapse/steroid therapy and by excluding Gd-enhancing lesions from the analysis. Moreover, there was some selection bias as only newly diagnosed patients or patients with longer disease duration were included. For that reason, we performed univariate analysis instead of linear regression, as it may have affected the interpretation of data. We also did not evaluate the differences in spatial localization of different types of lesions as well as their volumes which we aim to address in the future. Finally, we did not consider gray matter or spinal cord atrophy, which is also associated with clinical disability.

We confirm that PRLs are more destructive than both DSHLs and SILs, leading not only to intralesional tissue destruction but also to diffuse periplaque WM damage in comparison to other types of lesions. Since the extent of retrograde neuroaxonal damage could be reliably and rapidly assessed using MDME-based synthetic MRI, this method may be well applicable in the early adjustment of MS treatment in clinical routine setting and/or clinical trials in the future. Thus, our results again underscore the clinical relevance of detecting and targeting PRLs in the future to eventually slow down disability progression in pwMS.

## Supplemental Material

sj-docx-1-msj-10.1177_13524585231197954 – Supplemental material for Paramagnetic rim lesions lead to pronounced diffuse periplaque white matter damage in multiple sclerosis Click here for additional data file. Supplemental material, sj-docx-1-msj-10.1177_13524585231197954 for Paramagnetic rim lesions lead to pronounced diffuse periplaque white matter damage in multiple sclerosis by Nik Krajnc, Victor Schmidbauer, Joel Leinkauf, Lukas Haider, Gabriel Bsteh, Gregor Kasprian, Fritz Leutmezer, Barbara Kornek, Paulus Stefan Rommer, Thomas Berger, Hans Lassmann, Assunta Dal-Bianco and Simon Hametner in Multiple Sclerosis Journal

sj-docx-2-msj-10.1177_13524585231197954 – Supplemental material for Paramagnetic rim lesions lead to pronounced diffuse periplaque white matter damage in multiple sclerosis Click here for additional data file. Supplemental material, sj-docx-2-msj-10.1177_13524585231197954 for Paramagnetic rim lesions lead to pronounced diffuse periplaque white matter damage in multiple sclerosis by Nik Krajnc, Victor Schmidbauer, Joel Leinkauf, Lukas Haider, Gabriel Bsteh, Gregor Kasprian, Fritz Leutmezer, Barbara Kornek, Paulus Stefan Rommer, Thomas Berger, Hans Lassmann, Assunta Dal-Bianco and Simon Hametner in Multiple Sclerosis Journal

sj-docx-3-msj-10.1177_13524585231197954 – Supplemental material for Paramagnetic rim lesions lead to pronounced diffuse periplaque white matter damage in multiple sclerosis Click here for additional data file. Supplemental material, sj-docx-3-msj-10.1177_13524585231197954 for Paramagnetic rim lesions lead to pronounced diffuse periplaque white matter damage in multiple sclerosis by Nik Krajnc, Victor Schmidbauer, Joel Leinkauf, Lukas Haider, Gabriel Bsteh, Gregor Kasprian, Fritz Leutmezer, Barbara Kornek, Paulus Stefan Rommer, Thomas Berger, Hans Lassmann, Assunta Dal-Bianco and Simon Hametner in Multiple Sclerosis Journal

sj-jpg-4-msj-10.1177_13524585231197954 – Supplemental material for Paramagnetic rim lesions lead to pronounced diffuse periplaque white matter damage in multiple sclerosis Click here for additional data file. Supplemental material, sj-jpg-4-msj-10.1177_13524585231197954 for Paramagnetic rim lesions lead to pronounced diffuse periplaque white matter damage in multiple sclerosis by Nik Krajnc, Victor Schmidbauer, Joel Leinkauf, Lukas Haider, Gabriel Bsteh, Gregor Kasprian, Fritz Leutmezer, Barbara Kornek, Paulus Stefan Rommer, Thomas Berger, Hans Lassmann, Assunta Dal-Bianco and Simon Hametner in Multiple Sclerosis Journal

sj-jpg-5-msj-10.1177_13524585231197954 – Supplemental material for Paramagnetic rim lesions lead to pronounced diffuse periplaque white matter damage in multiple sclerosis Click here for additional data file. Supplemental material, sj-jpg-5-msj-10.1177_13524585231197954 for Paramagnetic rim lesions lead to pronounced diffuse periplaque white matter damage in multiple sclerosis by Nik Krajnc, Victor Schmidbauer, Joel Leinkauf, Lukas Haider, Gabriel Bsteh, Gregor Kasprian, Fritz Leutmezer, Barbara Kornek, Paulus Stefan Rommer, Thomas Berger, Hans Lassmann, Assunta Dal-Bianco and Simon Hametner in Multiple Sclerosis Journal

sj-jpg-6-msj-10.1177_13524585231197954 – Supplemental material for Paramagnetic rim lesions lead to pronounced diffuse periplaque white matter damage in multiple sclerosis Click here for additional data file. Supplemental material, sj-jpg-6-msj-10.1177_13524585231197954 for Paramagnetic rim lesions lead to pronounced diffuse periplaque white matter damage in multiple sclerosis by Nik Krajnc, Victor Schmidbauer, Joel Leinkauf, Lukas Haider, Gabriel Bsteh, Gregor Kasprian, Fritz Leutmezer, Barbara Kornek, Paulus Stefan Rommer, Thomas Berger, Hans Lassmann, Assunta Dal-Bianco and Simon Hametner in Multiple Sclerosis Journal
