# Supplementary material for: Paramagnetic rim lesions lead to pronounced diffuse periplaque white matter damage in multiple sclerosis
Source: Mult Scler. 2023 Sep 15;29(11-12):1406–17. doi: 10.1177/13524585231197954 (PMC10580674; doi:10.1177/13524585231197954)
Supplement: sj-docx-1-msj-10.1177_13524585231197954 – Supplemental material for Paramagnetic rim lesions lead to pronounced diffuse periplaque white matter damage in multiple sclerosis [file sj-docx-1-msj-10.1177_13524585231197954.docx]

**Figure S1.** Two paramagnetic rim lesions (PRLs) on synthetically generated T2-weighted contrasts (a and c) and the corresponding susceptibility-weighted imaging (SWI) data (b and d).

**Figure S2.** Two susceptibility-weighted imaging (SWI)-isointense lesions (SILs) on synthetically generated T2-weighted contrasts (a and c) and the corresponding SWI data (b and d).

**Figure S3.** Two lesions with diffuse susceptibility-weighted imaging (SWI)-hypointense signal (DSHLs) on synthetically generated T2-weighted contrasts (a and c) and the corresponding SWI data (b and d).
