# Supplementary material for: Paramagnetic rim lesions lead to pronounced diffuse periplaque white matter damage in multiple sclerosis
Source: Mult Scler. 2023 Sep 15;29(11-12):1406–17. doi: 10.1177/13524585231197954 (PMC10580674; doi:10.1177/13524585231197954)
Supplement: sj-docx-2-msj-10.1177_13524585231197954 – Supplemental material for Paramagnetic rim lesions lead to pronounced diffuse periplaque white matter damage in multiple sclerosis [file sj-docx-2-msj-10.1177_13524585231197954.docx]

**Supplementary Table 2.** MRI metrics in different MS phases.

|  | **All (n=30)** | **eRMS (n=10)** | **lRMS (n=11)** | **SPMS (n=9)** | ***p*-value**^§^ |
| --- | --- | --- | --- | --- | --- |
| **T1 relaxation time (ms)^a^** | | | | | |
| PRLs | **2030.5 (1519–2540)** | **1891 (1323.9–2575.3)** | **1869.3 (1491.4–2451.3)** | **2394.5 (2030.5–3040)** | **0.033** |
| PRL PPA | 926.5 (860.5–980.5) | 904.3 (793.3–958.5) | 919 (858.3–973.8) | 982 (927–1093.5) | 0.059 |
| DSHLs | **1615.8 (1403.3–1953.5)** | **1418.8 (1212.4–1734.1)** | **1790 (1421–2542.5)** | **1678.3 (1487.8–1987.1)** | **0.010** |
| DSHL PPA | **835.8 (793–873.9)** | **796.5 (773.6–841.3)** | **851.8 (793.9–885.9)** | **845.8 (822.6–903.9)** | **0.005** |
| SILs | 1199.5 (1089.6–1334.6) | 1202.3 (1113.9–1322.5) | 1209 (1089–1344) | 1138 (1077.5–1319) | 0.640 |
| SIL PPA | 780.3 (748.4–814.9) | 774.8 (734–796.4) | 781 (747.5–836.5) | 796 (764–820) | 0.244 |
| NAWM | 723.8 (706.9–787.3) | 723.5 (697.5–777.1) | 769 (708–827) | 711.5 (700–760) | 0.276 |
| **T2 relaxation time (ms)^a^** | | | | | |
| PRLs | **154 (120–192)** | **136 (112–188.5)** | **152.5 (118.1–176.4)** | **215.5 (150.5–342.5)** | **0.035** |
| PRL PPA | 87 (81.5–91.5) | 83.5 (79.6–90.1) | 87.8 (82.9–91.5) | 85 (83–106.5) | 0.583 |
| DSHLs | **136.8 (118.9–169.6)** | **116.5 (95.8–135.6)** | **148.5 (124.1–174.5)** | **147.8 (128.5–180.4)** | **0.001** |
| DSHL PPA | **79.8 (76.4–83.1)** | **76.8 (72.6–81)** | **78.8 (76.3–82)** | **82 (77.6–86.5)** | **0.002** |
| SILs | 102 (93.3–113.4) | 96.5 (90.9–108.8) | 102 (97–116) | 106 (92.5–119.5) | 0.099 |
| SIL PPA | 76.5 (72.5–80) | 76.3 (69.5–79.3) | 77 (73.5–80) | 76 (72.5–81) | 0.291 |
| NAWM | 64.8 (63–67.5) | 68 (62.9–69.6) | 64.5 (63.5–67) | 64 (62.5–66.5) | 0.209 |
| **PD metrics^a^** | | | | | |
| PRLs | 103.6 (98.2–107.1) | 103.3 (97.6–107.1) | 104.0 (98.3–107.4) | 104.2 (97.2–105.3) | 0.742 |
| PRL PPA | 70.7 (68.0–74.2) | 69.8 (67.4–73.4) | 70.1 (68.2–73.9) | 74.0 (71.8–78) | 0.111 |
| DSHLs | 97.8 (89.9–104.6) | 97.5 (88.9–103.9) | 101.8 (89.7–106.2) | 97.5 (89.7–101.8) | 0.427 |
| DSHL PPA | 67.4 (65.7–69.7) | 67.1 (65.8–69.1) | 67.8 (65.2–69.9) | 67.8 (66.2–70.5) | 0.770 |
| SILs | **84.3 (80.7–89.3)** | **87.5 (81.9–93.9)** | **84.7 (82–89.2)** | **82.6 (79–86)** | **0.012** |
| SIL PPA | 65.7 (63.8–67.3) | 65.2 (63.8–67.5) | 65 (63.4–66.9) | 66.4 (64.2–67.7) | 0.173 |
| NAWM | 63.1 (61.5–64.4) | 61.9 (60.6–64.4) | 63.9 (61.7–65.6) | 63.5 (62.0–64.5) | 0.581 |

DSHL: lesions with diffuse SWI-hypointense signal, eRMS: early relapsing multiple sclerosis, lRMS: late relapsing multiple sclerosis, NAWM: normal-appearing white matter, PD: proton density, PPA: periplaque area, PRL: paramagnetic rim lesion, RMS: relapsing multiple sclerosis, SIL: SWI-isointense lesion, SPMS: secondary progressive multiple sclerosis

^a^Median and interquartile range

^§^Kruskal-Wallis test
