# Supplementary material for: Paramagnetic rim lesions lead to pronounced diffuse periplaque white matter damage in multiple sclerosis
Source: Mult Scler. 2023 Sep 15;29(11-12):1406–17. doi: 10.1177/13524585231197954 (PMC10580674; doi:10.1177/13524585231197954)
Supplement: sj-docx-3-msj-10.1177_13524585231197954 – Supplemental material for Paramagnetic rim lesions lead to pronounced diffuse periplaque white matter damage in multiple sclerosis [file sj-docx-3-msj-10.1177_13524585231197954.docx]

**Supplementary Table 1.** MR protocol.

| **Sequence** | **Plane** | **Acquisition Time** | **TR (ms)** | **TE (ms)** | **TI (ms)** | **Voxel (mm)** | **FOV (mm)** | **Matrix (slices)** |
| --- | --- | --- | --- | --- | --- | --- | --- | --- |
| FLAIR (3D) | Sagittal | 04:24 | 4800 | 340 | 1650 | 1 x 1 x 1 | 250 x 250 x 180 | 252 x 251 x 360 |
| T2 (3D) | Sagittal | 01:45 | 3000 | 280 | - | 1 x 1 x 1 | 250 x 250 x 180 | 252 x 251 x 360 |
| T1 (3D)^a^ | Sagittal | 05:45 | 11.43 | 5.27 | - | 0.7 x 0.7 x 0.7 | 256 x 240 x 180 | 368 x 345 x 257 |
| DWI (2D)^b^ | Axial | 00:49 | 4052.76 | 83.22 | - | 2.05 x 2.56 x 4 | 230 x 230 x 144 | 112 x 90 x 36 |
| SWI (2D) | Axial | 03:20 | 31 | 7.2 | - | 0.6 x 0.6 x 2 | 230 x 189 x 143 | 384 x 315 x 143 |
| MDME (2D) | Axial | 06:15 | 4529.15 | 100; 12.5 | - | 0.7 x 0.85 x 4 | 230 x 189 x 149 | 328 x 219 x 30 |

^a^Pre- and post application of *Gadobutrol* (Gadovist ®): 0.1 ml/kg bodyweight

^b^*b* = 0/1000 s/mm^2^

DWI: Diffusion-weighted imaging, FLAIR: Fluid-attenuated inversion recovery, FOV: Field-of-view, MDME: Multi-dynamic multi-echo, SWI: Susceptibility-weighted imaging, TE: Echo time, TI: Inversion time, TR: Repetition time
