# Supplementary figures and images for: Paramagnetic rim lesions lead to pronounced diffuse periplaque white matter damage in multiple sclerosis
Source: Mult Scler. 2023 Sep 15;29(11-12):1406–17. doi: 10.1177/13524585231197954 (PMC10580674; doi:10.1177/13524585231197954)

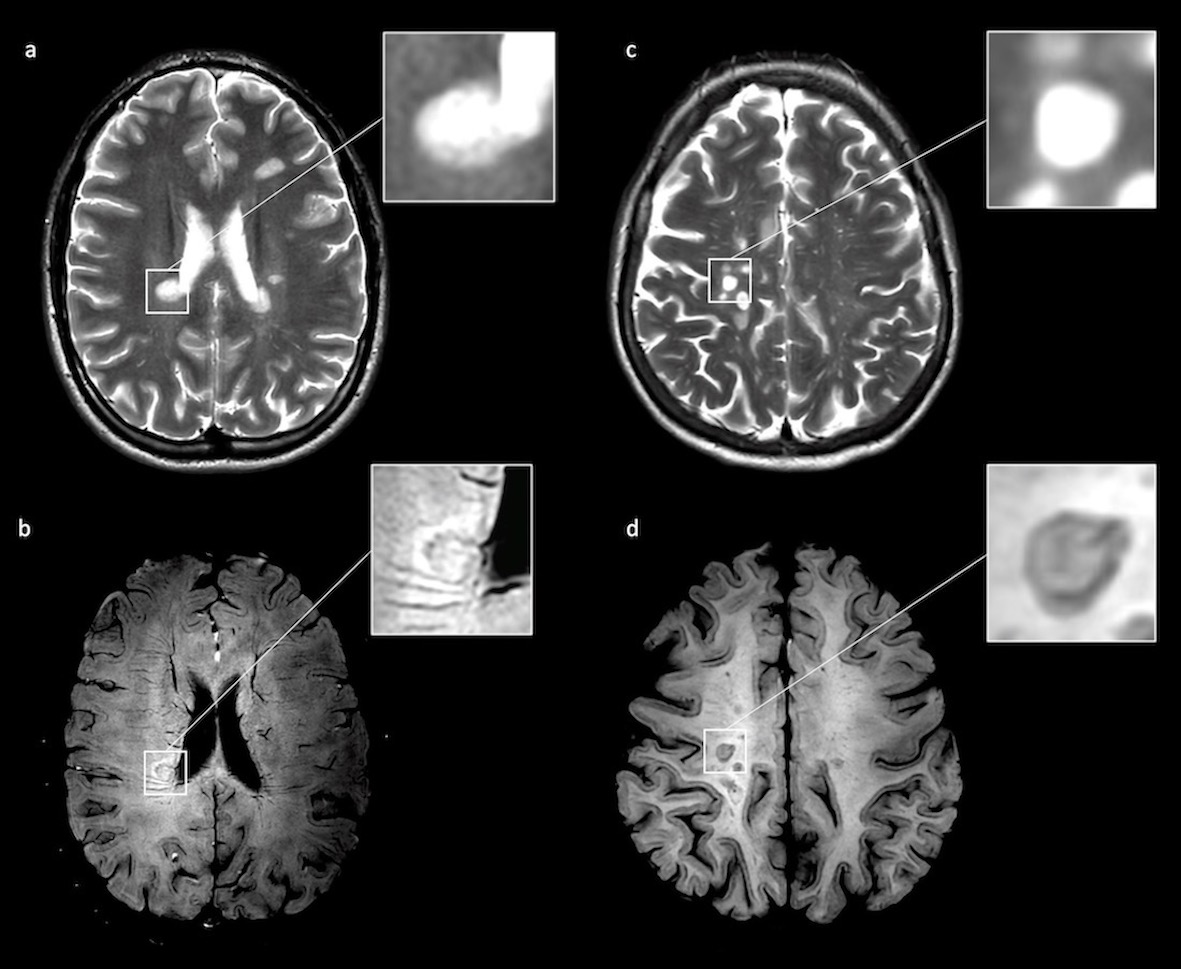

Supplement: sj-jpg-4-msj-10.1177_13524585231197954 – Supplemental material for Paramagnetic rim lesions lead to pronounced diffuse periplaque white matter damage in multiple sclerosis [file sj-jpg-4-msj-10.1177_13524585231197954.jpg]

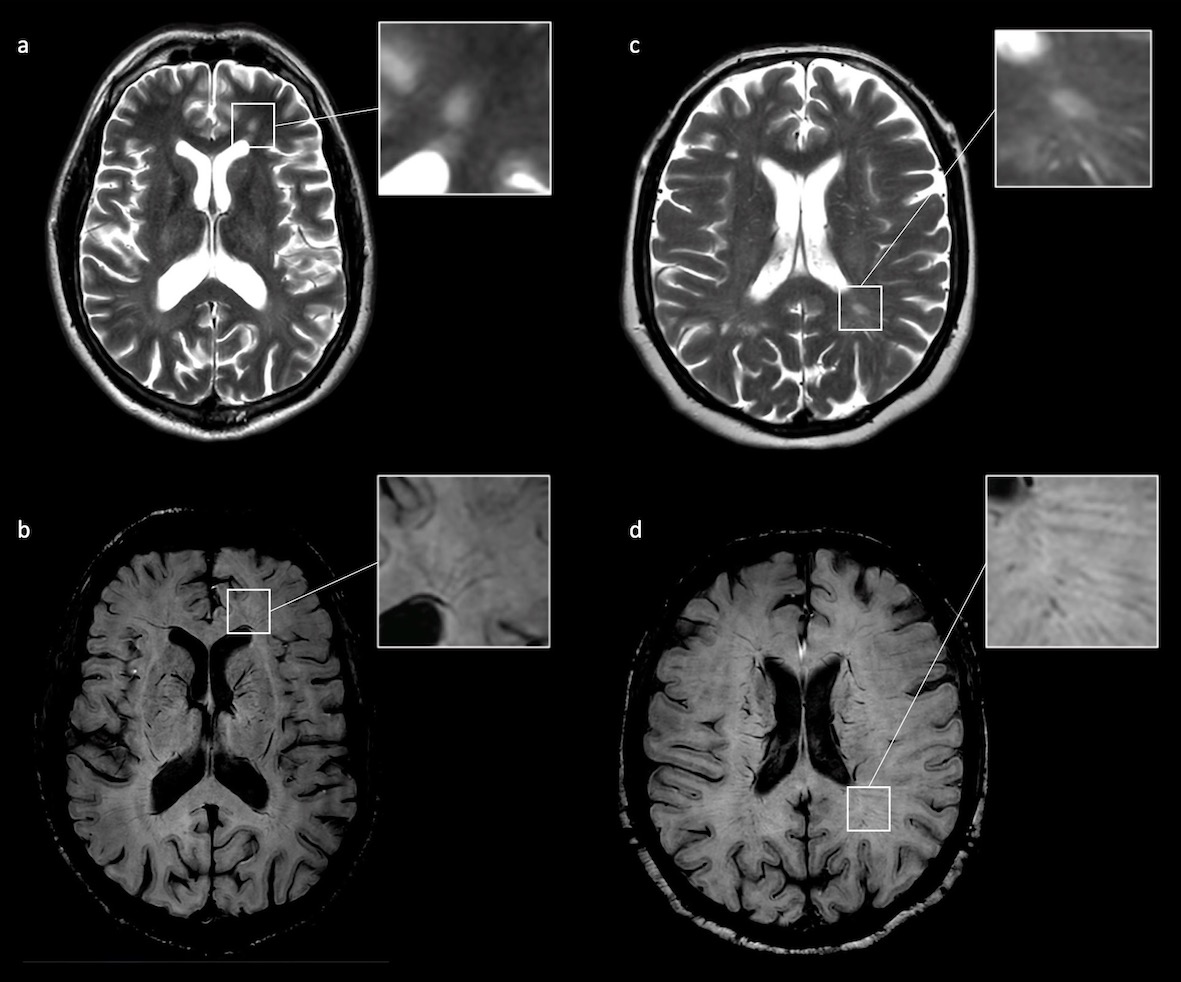

Supplement: sj-jpg-5-msj-10.1177_13524585231197954 – Supplemental material for Paramagnetic rim lesions lead to pronounced diffuse periplaque white matter damage in multiple sclerosis [file sj-jpg-5-msj-10.1177_13524585231197954.jpg]

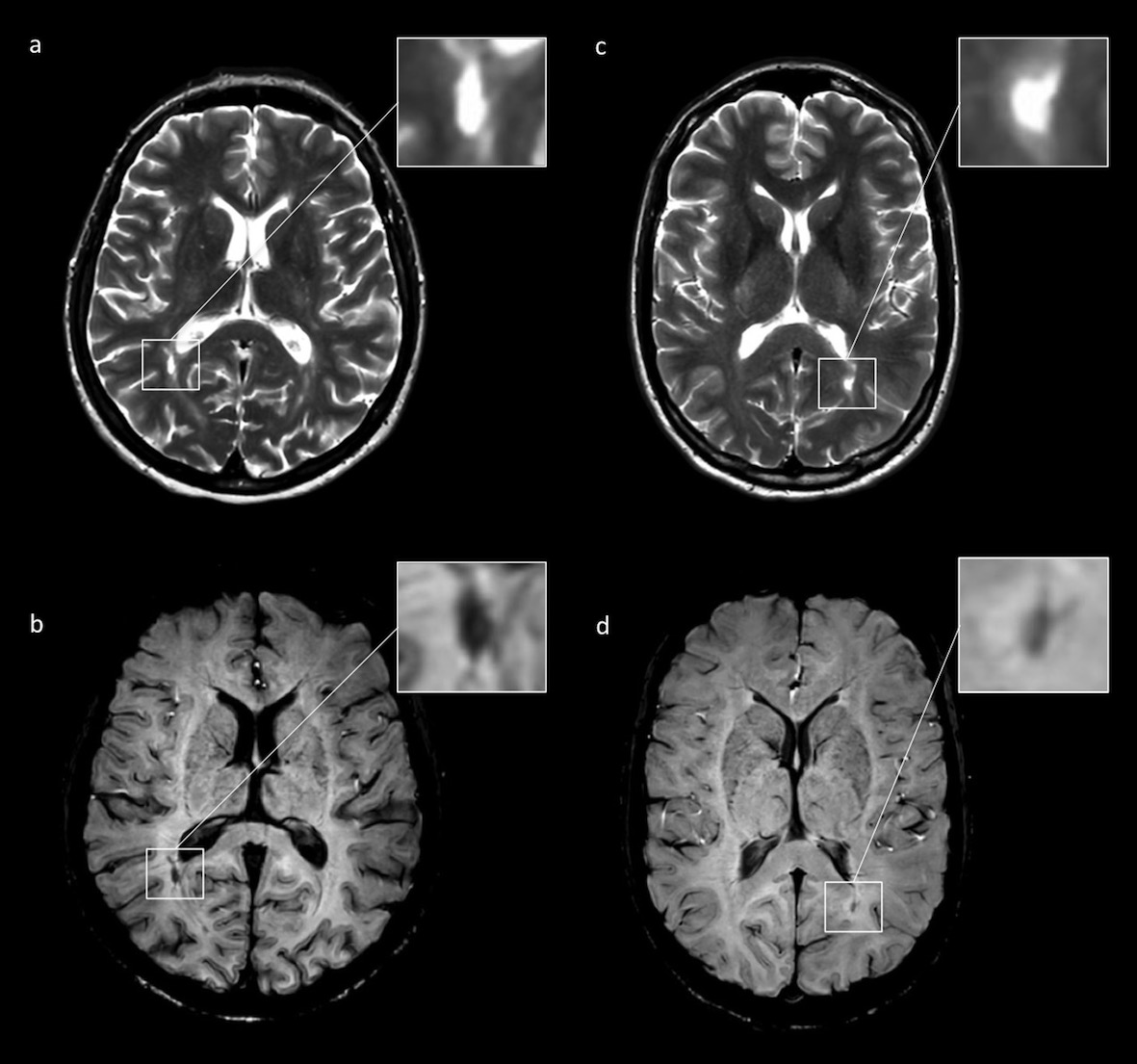

Supplement: sj-jpg-6-msj-10.1177_13524585231197954 – Supplemental material for Paramagnetic rim lesions lead to pronounced diffuse periplaque white matter damage in multiple sclerosis [file sj-jpg-6-msj-10.1177_13524585231197954.jpg]
